# Series: Public engagement with research. Part 2: GPs and primary care researchers working inclusively with minoritised communities in health research to help address inequalities

**DOI:** 10.1080/13814788.2024.2322996

**Published:** 2024-03-13

**Authors:** Yumna Masood, Anica Alvarez Nishio, Bella Starling, Shoba Dawson, Jon Salsberg, Steven Blackburn, Esther van Vliet, Carina A.C.M Pittens

**Affiliations:** a Centre for Evidence Based Medicine | Nuffield Department of Primary Care Health Sciences |, University of Oxford Radcliffe Primary Care Building, University of Oxford, Oxford, UK; b Public Contributor, London, UK; c Manchester University NHS Foundation Trust, Manchester, UK; d Centre for Academic Primary Care, University of Bristol, Bristol, UK; e University of Limerick, Family Medicine Limerick, Limerick, Ireland; f Institute of Applied Health Research, University of Birmingham, Birmingham, UK; g Academic Collaborative Centers, Knowledge Transfer Office, Tilburg University, Tilburg, The Netherlands; h Athena Institute, Vrije Universiteit Amsterdam, Amsterdam, The Netherlands

**Keywords:** Public engagement, health inequalities, minoritised communities, primary care

## Abstract

Public engagement in health research is vital for addressing health disparities and promoting inclusivity among minoritised communities who often face barriers to accessing healthcare. Minoritised communities are groups, which have been made minorities by a dominant culture, race, ethnic group and/or social class and may experience health inequalities as a result. By incorporating diverse perspectives and lived experiences of minoritised communities, this approach aims to achieve contextually relevant research outcomes that reduce health inequalities and improve overall well-being. However, underrepresentation and lack of inclusivity challenges persist, necessitating the establishment of inclusive partnerships and grassroots participatory methodologies.

To foster inclusive public engagement, it is important to overcome structural and cultural barriers, address socioeconomic challenges, and build trust with minoritised communities. This can be achieved by promoting a cultural shift that values inclusivity, providing comprehensive training to researchers, and collecting rigorous data on engagement demographics for transparency and accountability. Involving minoritised communities in decision-making through participatory research approaches enhances trust and yields successful outcomes. Additionally, allocating sufficient resources, collaborating in co-production, and prioritising the diverse needs and perspectives of stakeholders contribute to fostering inclusive public engagement in research.

Overall, inclusive engagement practices particularly in primary care research have the potential to reduce health inequalities and cater to the unique requirements of minoritised communities, thereby creating more impactful outcomes and promoting equitable healthcare access.

## KEY MESSAGES

There is an important need to engage with minoritised communities in primary care research

Engaging diverse communities in research helps produce relevant research to address health inequalities.

The exclusion of minoritised communities from research can be addressed by taking action towards more inclusive engagement.

## Introduction

There is growing evidence of inequalities in access to and outcomes from primary care [1]. As consideration of equality, diversity and inclusion (EDI) in healthcare becomes a growing global priority, GPs and primary care researchers are uniquely placed to address the challenges of inequality experienced by many communities.

Minoritised individuals and communities can be defined as those ‘whose collective cultural, economic, political and social power has been eroded through the targeting of identity in active processes that sustain structures of hegemony’ [2]. ‘Minoritised’ is not a simple concept. It portrays the systematic and societal structures and processes that place different sets of people and communities in inequitable places. Communities can be minoritised due to individual factors (e.g. physical or mental abilities), structural factors (e.g. a person’s location in a hierarchical socio-cultural society), personal circumstances (e.g. lifestyle choices, geographical location, education and literacy) and (unconscious) bias in those who make decisions [2]. For example, being overweight and obese are more prevalent among adolescents with a low socioeconomic position Intersectionality – the complex interplay of these different forms of inequality and the how this shapes people’s unique experience of and access to healthcare is increasingly recognised [3, 4]. Amplified by the COVID-19 pandemic, addressing these inequalities is a challenge faced by primary care clinicians and researchers.

As the second of a four-part series, this article builds upon the fundamentals of public engagement in research (Part 1) by discussing the importance of engaging with minoritised communities [5], who experience health inequalities, in primary care research. We provide rationale for public engagement with these communities, surfaces barriers to engagement, and suggests some potential mitigation strategies. We aim to assist GPs and primary care researchers with strategies to improve the inclusivity of research as a means to address health inequalities.

## Tackling health inequalities through engagement with minoritised communities in research

Engaging with minoritised communities, as users of primary care research, is seen as crucial in (a) preventing or overcoming health inequalities [6]; (b) ensuring that research is generalisable, contextualised and needs-oriented, and therefore viewed by minoritised communities as legitimate; and (c) that it does not perpetuate existing inequalities but rather benefits all of society [7]. Public engagement is used to describe the many ways that people contribute their views and personal lived experience (e.g. of a condition, of living with a protected characteristic) to help prioritise, plan, deliver, evaluate and disseminate health and social care research [5]. One approach is through the use of ‘participatory methodologies.’ This refers to research that involves the active and meaningful participation of community members throughout the research process, aiming to establish equitable partnerships between researchers and communities [8]. These methodologies emphasise collaboration, shared decision-making, and community expertise and knowledge recognition. Participatory methodologies promote the inclusion of diverse perspectives, ensure that community voices are heard and valued, and prioritise the empowerment of communities in shaping research agendas and outcomes [9].

Though inclusive engagement and participatory methodologies are no guarantee for reducing health inequalities, they can be a means of broadening representation and ensuring that diverse perspectives and experiences are taken into account in the research process, and they can work to break down barriers, such as suspicion and stigma [10].

However, studies often neglect to identify issues of equality, diversity and inclusion, which may in turn be barriers (or enablers) to engagement [11]. Full, inclusive, effective engagement is challenging to achieve. As a result, minoritised people and communities are often underrepresented in health research.

## Barriers to inclusive public engagement in research

Public engagement in research can provide various benefits to the process and outcomes of research [5]. Yet, numerous barriers can arise even where there is an understanding of the need and a commitment to invest in inclusive approaches.

Acknowledging these challenges is the first step in determining how to address them effectively. Following is a discussion of three broad categories of barriers to participation followed by a discussion of some strategies to mitigate against these and promote inclusivity.

### Structural and research-collaboration-related barriers

Minoritised communities are often excluded due to primary care research processes and cultural misunderstandings. Some barriers occur because of the research process itself. Commonly used formats – such as advisory boards, focus group discussions and questionnaires – can be inappropriate for some minoritised communities [12]. There may be a lack of resources for developing appropriate study materials (multi-lingual, large format, etc.) or a lack of staff with the skills, creativity and expertise to work with particular minoritised communities [13]. Another barrier is that researchers may have a poor understanding of the specific needs of minoritised communities (such as communication, support and accessibility needs), may not bring communities into research at the planning stages, and may need to develop new approaches. If conversations with minoritised communities have not occurred at the earliest stage, later conversations will not address core needs. Even attempts at increasing inclusivity may inadvertently raise barriers. By identifying people to collaborate with through ‘gatekeeper’ organisations (healthcare bodies, patient groups, charities, etc.), researchers often reach individuals who already are interested and engaged rather than accessing new voices: those who are not in contact with any (in)formal organisation [14].

### Logistical and economic barriers

At an even more basic level, there are people from minoritised communities who, due to their socioeconomic situation, cannot afford to participate in research [15]. Although some honoraria are offered, these rarely reflect the true participation costs. People who are part of engagement activities may incur direct costs (e.g. childcare, transportation), indirect costs (the perceived need to buy something ‘decent’ to wear) and opportunity costs (missed work, social events, medical appointments).

An anecdotal example:

*A woman with multiple long-term conditions who lives in a rural village would like to participate in research. While a project might pay for her time and bus fare, it cannot compensate the neighbour who must take time off work to drive the woman to the bus stop that would otherwise be inaccessible. Nor can it resolve the problem of the woman being offered a clinical appointment at short notice; she must choose between cancelling her attendance at the research event or having a negative mark on her medical record for having a ‘declined appointment’ and risk not being offered another one.*

### Barriers related to trust and power dynamics

Lack of trust between researchers and minoritised communities also hinders engagement practices [12]. Mistrust can have multiple causes: minoritised people, especially those with a migration background, can have negative historical experiences with authority, fear surveillance or think that participation may negatively affect their asylum application [14]. Addressing trust barriers to engagement is especially relevant for primary care since GPs are the first point of contact for many minoritised communities. GPs are often considered a trusted point of contact where mistrust may impede communities’ access to care or engagement in public health initiatives. The work of GPs in encouraging engagement may help mitigate this [16].

Another significant barrier is the connection between distrust and public misunderstanding about the mechanisms and utility of research. One reason for this may be that some research topics can be considered as ‘common knowledge’ to the public. This may give rise to suspicion of the researchers’ ‘real’ motives for ‘researching’ something the public considers self-evident, leading to distrust in the researchers, the research and the process:

An anecdotal example:

*A research team looked into why mothers get a first vaccination for their children but no follow-ups. Lived experience showed it is because, in addition to being unable to afford to take multiple days off work, in households with several children, a mother cannot simultaneously take one child to the doctor and another to school and will get penalised if the second child is absent. In the public domain this is considered to be something ‘everyone knows.’ However, researchers, who may have flexible work schedules and greater authority or confidence when negotiating with schools, may not experience this in their lives, so they do not identify systemic barriers, and instead, misattribute it as mothers’ lack of conscientiousness. Surfacing this knowledge enabled researchers to make impactful changes in protocol and policy, leading to higher uptakes of vaccines – but to the public, the crucial piece of knowledge was self-evident.*

Distrust around sharing or misusing personal information and other data protection issues is also higher in ethnic groups [17].

Power dynamics can also be a barrier. As seen in the vaccination anecdote above, researchers and minoritised communities have relative differences in their agency, capacity, knowledge and access to resources [18]. This can lead to the public not seeing research as a priority compared to other more pressing concerns in their lives and limiting their ability to participate in research. Another example would be public members attending workshops but being too intimidated by researchers or formal processes to speak up or telling researchers what they think the researchers want to hear.

Further explorations regarding the power dynamics within public engagement will be addressed in the third article of this series [citation to be included when published]

## Strategies to improve inclusivity in research

Though the barriers are extensive, GPs and primary care researchers can adopt various strategies for addressing them. While mandated top-down approaches can establish a framework for inclusivity, we encourage researchers to proactively adopt grassroots, bottom-up approaches, including participatory methodologies, to establish inclusive partnerships between researchers and communities. Below, we describe some strategies – coming from practice or found in literature – to give practical examples of addressing barriers. We present case studies of successful engagement practices with minoritised communities in three boxes. Key factors across all these cases are (a) adapting strategies to specific contextual demands, (b) using participatory approaches from the earliest point – when ideas are being generated – and (c) continuing to build and maintain trusting relationships throughout, and ideally beyond, the end of a project.

### Strategies to stimulate a shift in research culture

Culture change is complex and progressive and will include many factors. One factor towards culture change is to acknowledge specific moments when individuals can act as levers of change. Very often, GPs are the first and most consistent point of contact for minoritised communities [19]. This continuity and GPs’ authority can be crucial in engendering trust. GPs can leverage this unique role by discussing with their patients the importance of research and how public engagement assists in producing research of value.

An anecdotal example:

*‘Migrants are rarely perceived as people who can contribute to society in terms of solving problems – they are often seen as groups that are a problem, making it difficult to persuade them that their voices matter. So, we [researchers] had to build people’s confidence and reassure them that their experiences of language and cultural barriers in GP consultations were vital and necessary to the research. We explained that they represented a critical stakeholder group, and we needed them on board because their voices are often missing in research about health policies, which directly affect their lives. Most importantly, we developed strong trust relationships – this meant they could tell us the truth from their perspective, and we would respect it.’[20]*

This anecdote shows that to enable culture change, GPs, researchers and the public need to confront their prejudices and their limited knowledge of what people in different circumstances value.

However, confronting presumptions is not enough. Culture change requires complementary approaches. One practical strategy is for organisations to provide researchers with adequate learning and development. It is also important that training is meaningfully co-developed with a range of stakeholders, including members of minoritised communities, and that it goes beyond first-order training in ‘unconscious bias’ through ideas of ‘cultural safety’ to concerns of specific relevance to the communities and research at hand [21]. (A list of training material for primary care researchers to support inclusive engagement sourced from the United Kingdom is provided in Supplementary File 1. It is also important to note that professional development in public engagement is not seen as a one-off event and that adequate resources are available to sustain learning.

Training alone, however, is not effective in shifting research culture. Another approach is to increase the range and type of data collected on diversity characteristics of study participants. While acknowledging (as discussed above) that some minoritised communities are sceptical of the purposes of data collection, collecting rigorous data can support data-driven approaches to culture change: collecting, analysing and reflecting on data on diversity over time can increase accountability and transparency and engender trust [22].

Currently there is little obligation for research studies to record ethnicity and broader demographic indicators routinely. This limits analysis as to whether the research being carried out is relevant to those with poorer health outcomes. While there are no rules for mandatory inclusion across demographic indicators, some research funders and journals are beginning to acknowledge the importance of collecting this data. Furthermore, careful consideration of recording personal information from minoritised communities sensitively and appropriately is needed.

### Strategies to stimulate shift in research collaboration

Beyond adapting research *culture*, it is also important to address research collaboration approaches. This includes conceiving and adopting regional, strategic and ‘whole-system’ strategies to working in partnership with people in research [23].

One structural strategy is re-examining who determines which research should be done (agenda-setting) and how [7]. In one example of a concrete strategy to shift research collaboration with communities in the U.K., the National Institute of Health and Care Research (NIHR) Programme Grants for Applied Research (PGfAR) recently launched an innovative funding stream to encourage communities to generate ideas for research, in partnership and aided by the academic community [24]. Cases studies giving examples of other strategies and approaches to foster research collaboration with minoritised communities in Canada, the Netherlands and the U.K. can be found in Boxes 1–3, respectively.

Box 1. Case study: Participatory approaches.

*The Kahnawake Schools Diabetes Prevention Project (KSDPP.org) is a longstanding collaboration between university-based researchers and community members from an Indigenous community in Canada [28]. It was formed in 1994 following the presentation of alarming research results to the community about the incidence and prevalence of Type 2 diabetes and its complications. When the community told the researchers – who were family doctors (GPs) working in the local hospital – to ‘do something about it so that future generations would not bear this burden,’ the family doctors decided that the only way a solution would work was if the community itself led the effort.*

*This led to the creation of a community-based participatory primary prevention project aimed at increasing healthy behaviour and ultimately lowering rates of Type 2 diabetes. The project is governed by a community advisory board on which only community members have a vote and approve all intervention activities, research protocols and research dissemination].*

*Community ownership of the research process has built long-lasting trust between the community and academic researchers (still ongoing after nearly 30 years!), and built capacity within the community (training of lay health workers as well as Indigenous academic MSc and PhD researchers) (KSDPP.org), led to a permanent intervention presence in the community schools, and produced over 60 scientific publications.*

Box 2. Case study: Using Participatory Action Research (PAR) to overcome practical barriers.

*In 2013, the city council of Amsterdam, the Netherlands, started the Amsterdam Healthy Weight Approach programme [29]. The programme focuses on children and adolescents with the highest risks of developing poorer health outcomes, such as being overweight and obese. These children and adolescents often grow up in families with a low socioeconomic position (SEP) and have a migration background. The programme coordinated various preventative activities. There was cooperation across all departments of the City of Amsterdam and with third* ***parties such as schools,*** *voluntary organisations, welfare, (youth) health and social care providers, a health insurer, retailers, and academics.*

*In one study, adolescents with a low SEP have been engaged through a participatory action research (PAR) approach to develop tailored health promotion material and learn about a healthy lifestyle [29]. The PAR approach turned out to be successful in engaging this underserved community. To better align with the girls, activities – facilitated by female researchers – varied greatly and were based on the girls’ suggestions, e.g. cooking workshops and making a healthy lifestyle a more attractive choice for other adolescents.*

*Moreover, time and location depended on the girls’ motivations and agendas. Consequently, the girls experienced the research activities as fun and were actively engaged. Active engagement led to higher acceptability of the activities, increased empowerment, and a sense of ownership. Through the co-creation process, the girls learned much about a healthy lifestyle and expressing their own opinion. Policymakers and health promotors became more aware of the girls’ complex needs regarding a healthy lifestyle.*

Box 3. Case study: ‘We are not hard to reach, but we may find it hard to trust’ – Understanding co-production.

*Social innovation approaches, including sandpit events, seek to build and level relationships between different groups. This ‘sandpit’ event promoted new conversations between health researchers and people from diverse and marginalised groups. It aimed to shift the power balance and to encourage and fund innovations suggested by community members. In a joint venture between the Greater Manchester Black and Minority Ethnic (GMBME) Network, a public involvement team (Vocal), artists and several researchers, community members designed and pitched their own ideas for research projects. Everyone at the workshop voted, and the top six projects received funding. The workshop encouraged participants to build mutual trust, express their views and, in this way, uncover innovative health solutions [30].*

To be effective, though, such inclusive public engagement strategies require extra time and resources: to train researchers, to hire translators, for the extra effort needed to recruit from minoritised communities and to support new ways of working [12]. As noted above, the true costs to individuals are often not understood nor budgeted for adequately. Sensitive and realistic discussions need to occur to determine the true costs of participatory methods and approaches to inclusivity for inclusion in research grant proposals [25].

### Strategies to build trust

Trust is multidimensional, and it is essential to consider how to develop ways for minoritised communities and researchers to trust each other [26]. One way to build trust is to collaborate with community leads, advisors and advocates, or community outreach and education groups in a time and place and at the pace of their choosing (see Box 3) – which may not always be convenient for researchers or fit into traditional working practices or timings. Another is to co-produce with communities culturally sensitive materials and initiatives that emphasise personal and community-wide benefits [27]. A third way is evidence your trustworthiness by acknowledging shortcomings in the inclusiveness of research processes and procedures and putting in concrete actions to address them. Publicly committing to improving inclusion and addressing systemic barriers to engagement with community members’ input will in turn have built trust with sceptical community groups.

## Conclusion

Primary care is believed to be a place where health inequalities can be reduced [1]. GPs are often a first and trusted point of contact for minoritised communities. As such, there is an importance for research in this area to ensure it actively contributes to the reduction of health disparities through the inclusion of minoritised communities in ways that are contextually appropriate to develop outcomes that address health inequalities. Public engagement improves trust, ensures research directly addresses public needs and reduces inequalities. However, there are numerous systemic inequalities in research and many practical barriers to engagement with minoritised communities. Taking an active, participatory approach and forthrightly and sensitively addressing these issues will improve the inclusiveness of public engagement in research and by extension the delivery of care. To work successfully with minoritised communities, trust must be built incrementally over an extended period, trustworthiness must be shown, and practical steps must be taken to ensure true inclusivity. While more research is needed on the various forms of value public engagement brings, by working together more inclusively, from idea development to decision making to dissemination and delivery, not only will engagement be contextually appropriate but it also will lead to more rigorous, insightful research and beneficial, translational outcomes.

## Supplementary Material

Supplemental Material
