# Supplementary material for: Series: Public engagement with research. Part 2: GPs and primary care researchers working inclusively with minoritised communities in health research to help address inequalities
Source: Eur J Gen Pract. 2024 Mar 13;30(1):2322996. doi: 10.1080/13814788.2024.2322996 (PMC10939099; doi:10.1080/13814788.2024.2322996)
Supplement: Supplemental Material [file IGEN_A_2322996_SM7334.docx]

Supplementary File 1. – List of training resources to support inclusive public engagement in research

| Training | Location |
| --- | --- |
| Health Inequalities Assessment Toolkit | <https://www.hiat.org.uk/> |
| Inclusive Research online learning | <https://catalogue.manchester.ac.uk/browse/i3hs/open-courses/courses/inclusive-research> |
| Being Inclusive in Public Involvement: Guidance for Researchers | <https://www.nihr.ac.uk/documents/being-inclusive-in-public-involvement-in-health-and-care-research/27365> |
| NIHR Research Design Service EDI toolkit | <https://www.rdsresources.org.uk/edi-toolkit> |
| NIHR INCLUDE Resources  NIHR INCLUDE Ethnicity Framework | <https://sites.google.com/nihr.ac.uk/include/home/resources>  <https://www.trialforge.org/trial-forge-centre/include/> |
| NIHR toolkit for increasing participation of Black, Asian and minority ethnic groups in health and social care research | <https://arc-nenc.nihr.ac.uk/wp-content/uploads/2020/09/Toolkit-for-increasing-participation-of-Black-Asian-and-Minority-Ethnic-BAME-Groups-in-health-and-social-care-research.pdf> |
| INVOLVE: A Practical Guide to Being Inclusive in Public Involvement in Health Research - lessons from the Reaching Out programme | <https://www.invo.org.uk/wp-content/uploads/2020/03/INVOLVE_RO_report_FINAL_180220.pdf> |
| NIHR. Supporting equity and tackling inequality: how can NIHR promote inclusion in public partnerships. 2022. | <https://www.learningforinvolvement.org.uk/?opportunity=supporting-equity-and-tackling-inequality-how-can-nihr-promote-inclusion-in-public-partnerships> |
| NIHR Race Equality Framework | https://www.nihr.ac.uk/documents/nihr-race-equality-framework/30388 |
